# Improvement of Poly(lactic acid)-Poly(hydroxy butyrate) Blend Properties for Use in Food Packaging: Processing, Structure Relationships

**DOI:** 10.3390/polym14235104

**Published:** 2022-11-24

**Authors:** Mitul Kumar Patel, Marta Zaccone, Laurens De Brauwer, Rakesh Nair, Marco Monti, Vanesa Martinez-Nogues, Alberto Frache, Kristiina Oksman

**Affiliations:** 1 Division of Materials Science, Department of Engineering Sciences and Mathematics, Luleå University of Technology, SE-97187 Luleå, Sweden; 2 Proplast, Via Roberto di Ferro 86, 15122 Alessandria, Italy; 3 Bio Base Europe Pilot Plant (BBEPP), Rodenhuizekaai 1, 9042 Gent, Belgium; 4 Tecnopackaging, Polígono Industrial Empresarium, Calle Romero 12, 50720 Zaragoza, Spain; 5 Department of Applied Science and Technology and Local INSTM Unit, Politecnico di Torino, Viale Teresa Michel 5, 15121 Alessandria, Italy; 6 Mechanical & Industrial Engineering (MIE), University of Toronto, Toronto, ON M5S 3G8, Canada; 7 Wallenberg Wood Science Center (WWSC), Luleå University of Technology, SE-97187 Luleå, Sweden

**Keywords:** poly(lactic acid), poly(hydroxybutyrate), film blowing, cellulose nanocrystals, chitin nanocrystals, liquid-assisted extrusion, biodegradability, oxygen barrier properties

## Abstract

Poly(lactic acid)-poly(hydroxybutyrate) (PLA-PHB)-based nanocomposite films were prepared with bio-based additives (CNCs and ChNCs) and oligomer lactic acid (OLA) compatibilizer using extrusion and then blown to films at pilot scale. The aim was to identify suitable material formulations and nanocomposite production processes for film production at a larger scale targeting food packaging applications. The film-blowing process for both the PLA-PHB blend and CNC-nanocomposite was unstable and led to non-homogeneous films with wrinkles and creases, while the blowing of the ChNC-nanocomposite was stable and resulted in a smooth and homogeneous film. The optical microscopy of the blown nanocomposite films indicated well-dispersed chitin nanocrystals while the cellulose crystals were agglomerated to micrometer-size particles. The addition of the ChNCs also resulted in the improved mechanical performance of the PLA-PHB blend due to well-dispersed crystals in the nanoscale as well as the interaction between biopolymers and the chitin nanocrystals. The strength increased from 27 MPa to 37 MPa compared to the PLA-PHB blend and showed almost 36 times higher elongation at break resulting in 10 times tougher material. Finally, the nanocomposite film with ChNCs showed improved oxygen barrier performance as well as faster degradation, indicating its potential exploitation for packaging applications.

## 1. Introduction

Petroleum-based plastics are currently the most popular choice for packaging applications because of their low cost, ease of processability, and tunable properties, which can be modified to meet the specific needs of the products [1]. The most popular polymers used in the packaging industry are polyethylene (PE), polypropylene (PP), polyethylene terephthalate (PET), and polystyrene (PS), which together account for more than 90% of the total volume of plastics used in food packaging [2]. Traditional thermoplastics used in packaging are often made from non-renewable fossil fuels. In addition, most fossil fuel-based polymers are non-biodegradable; as a result, poor waste management contributes to several environmental issues, including high amounts of plastic waste in the environment, the emissions of toxic gases during the incineration of polymers, and the shortage of landfill space [3]. Thus, it is essential to replace these with sustainable and biodegradable polymers.

Among biopolymers from renewable resources, polylactic acid (PLA), is a potential candidate to replace petroleum-based non-biodegradable polymers due to its good mechanical performance (stiffness and strength) [4], transparency, excellent printability [5], processability [6], and economic feasibility compared to many other biodegradable polymers. However, despite the mentioned advantages, PLA has some disadvantages, which consequently limit its use in food packaging applications. These are its inherent brittleness, which is also reflected in the difficulty of producing thin-blowing films, and its moderate gas barrier performance [7].

Gas barrier properties are critical when considering a polymer for food packaging applications because many food products are sensitive to oxidation; hence, packages with low oxygen permeability are preferred. It is well known that the crystalline phase has a significant influence on the oxygen barrier properties of a material; as a consequence, increasing the crystallinity of PLA is required for food packaging applications. To enhance the crystallinity, blending PLA with other more crystalline biopolymers, such as poly(hydroxy alkanoates) (PHAs), has subsequently received a lot of attention in the food packaging industry. The most common representative of PHAs is poly(hydroxybutyrate) (PHB) [8] which has been extensively researched for its potential applications in food packaging [9]. Our previous research demonstrated that blending PLA with 25 wt% of PHB resulted in the formation of small, finely dispersed, and highly crystalline PHB spherulites in the PLA matrix [10]. This resulted in a significant increase in the crystallinity of the material, which ultimately improved its barrier performance. Likewise, Zhang et al. [11] reported that melt blending PLA with 25 wt% of PHB showed optimal miscibility between the two polymers.

Film blowing is the most widely accepted process for manufacturing polymer-based films in the packaging industry, such as barrier films (used to protect daily meat and vegetables), frozen food packaging, and shopping bags [12,13]. However, this processing is often challenging for both PLA and PHB because of their inherent brittleness, low melt strength, and reduced elongation as compared to PP and PE [14], resulting in unstable and wrinkled bubbles that tend to collapse during the film-blowing operations [15]. In addition, most food packaging applications require ductile properties and increased flexibility, which cannot be obtained with neat PLA:PHB blends. To address these concerns, different strategies have been proposed, such as the addition of plasticizer to improve the fluidity of the polymer blend [16,17], the use of viscosity enhancers to improve the material melt strength [18], or the development of nanocomposites by the introduction of nanomaterials such as chitin nanocrystals (ChNCs) or cellulose nanocrystals (CNCs) which concurrently improve the processability and the barrier properties of the produced films [15,19]. In this context, Herrera et al. [15] demonstrated the effectiveness of ChNCs in producing PLA-based nanocomposite blown films with improved processibility and mechanical properties.

Recently, cellulose and chitin nanocrystals have drawn the attention of researchers as promising reinforcing nanomaterials for producing bionanocomposites due to their large aspect ratios with good mechanical properties, as well as their biocompatible and renewable nature [20,21,22]. In addition to the improvement in mechanical properties, CNCs and ChNCs also act as nucleating agents for polymers, thereby enhancing the crystallization rate and increasing the crystallinity of the material. CNCs have been widely used as reinforcements to improve the thermal, mechanical, crystalline, and barrier properties of PLA [23] and PLA-PHB blends [19,24]. Singh et al. [25,26] showed that the addition of a small amount of ChNCs to PLA results in a significant improvement in PLA nucleus numbers due to faster crystallization speed and thus increased crystallinity. In addition, our previous study [10] demonstrated that the addition of 1 wt% ChNCs has a strong effect on the oxygen barrier properties of the PLA:PHB blend, which is generated simultaneously by the inherent barrier properties of ChNCs and the induction of a higher degree of crystallinity. Furthermore, ChNCs acted as an antistatic additive, resulting in less electrostatic interaction between the film surfaces, making it easier for the films to open [15]. Arrieta et al. [19] showed that the plasticized PLA-PHB-based bionanocomposite with 1 wt% functionalized CNC achieved higher elongation at break of about 150%, compared to the material without CNCs, showing comparable values to those of commercial stretchable films used for food packaging. In addition, Jandas et al. [27] reported that reactive extrusion of a PLA-PHB blend using MA (maleic anhydride) to form an interpenetrated network structure together with modified nanoclays resulted in excellent impact strength and high elongation at break.

Although CNCs and ChNCs have great potential as mentioned above, the hydrogen groups on the surface induce high attraction between the crystals, resulting in irreversible strong hydrogen bonds between them during preparation, which makes it difficult to achieve homogeneous dispersion of nanocrystals in polymers [28]. To avoid aggregation, nanocrystals generally undergo hydrophobic surface modifications before nanocomposite preparations [24,29,30]. Unfortunately, these hydrophobic surface modifications frequently have significant economic and environmental drawbacks, which limit their industrial potential [31]. Previously, a scalable liquid-assisted extrusion approach was proposed to improve the dispersion of nanocrystals and, as a consequence, improve the final properties of the final formulations [32,33,34]. According to this process, nanomaterials in aqueous form along with the dispersing aid are fed into polymer melt via extrusion. OLA has been considered an effective plasticizer for PLA [35] as well as for PLA-PHB blends [36,37]; additionally, it counteracts the inherent brittleness of PLA and PHB [38] and thereby improves the processability of the biopolymer and other properties required for food packaging applications. Hence, in this study, an oligomer lactic acid (OLA) was used as a compatibilizer and dispersing aid for CNCs and ChNCs in the manufacturing of bionanocomposite films.

The present study is the final phase of a pilot-scale project for the production of PLA-PHB-based bionanocomposite films by the film-blowing process. The aim of the study was to minimize the limitations of PLA films, which include processing difficulties, stiffness, brittleness, and low barrier properties, by incorporating PHB, nanoreinforcements (cellulose and chitin nanocrystals), and OLA with a focus on developing food packaging applications. In addition, we compared two different nanomaterials in different forms to be used as reinforcement for PLA-PHB: freeze-dried CNCs, which were added via conventional melt compounding, and ChNCs in an aqueous form, which was added as liquid dispersion into the melt compounding process. The processability of the materials and the morphological, thermal, and mechanical properties were studied, and the best films were selected for evaluation of their barrier and biodegradation behavior.

## 2. Materials and Methods

### 2.1. Materials

A commercial PLA, NatureWorks Ingeo Biopolymer 2003D, for fresh food packaging and tableware was purchased from Resinex Italy srl (Mornico al Serio, BG, Italy) and was used as the main biopolymer component in the biopolymer blend. Ingeo 2003D is an extrusion grade semicrystalline polymer with a melt flow index (MFI) of 6 g/10 min (measured at 210 °C and with a load of 2.16 kg), the tensile strength of 53 MPa, 3.5 GPa tensile modulus, and 6% elongation at break, as reported by the manufacturer.

A commercial polyhydroxyalkanoate PHB, IAM NATURE B6 E15, purchased from Gruppo MAIP srl (Settimo Torinese, TO, Italy) was used as the second component in the biopolymer blend. The PHB is an extrusion-grade biopolymer with an MFI of 3 g/10 min (measured at 170 °C and with a load of 5 kg), and the tensile strength of 20 MPa was reported by the manufacturer.

A biobased and biodegradable low-molecular-weight oligomer lactic acid (OLA, (Glyplast OLA2 purchased from Condensia Quimica SA, Barcelona, Spain) was used as a compatibilizer and processing aid. The OLA is a viscous liquid having a molecular weight of 500–600 g/mol, ester content >99%, a density of 1.10 g/cm^3^, and a viscosity of 90 mPa·s.

Microcrystalline cellulose (MCC) and shrimp chitin were used as starting materials for the production of the nanocrystals (CNCs and ChNCs) and were purchased from Glentham Life Sciences Ltd., Corsham, UK.

The used chemicals, sulfuric acid (H_2_SO_4_) and potassium phosphate (K_3_PO_4_), and sodium hydroxide (NaOH) used in the acid hydrolysis were purchased from Brenntag N.V. (Deerlijk, Belgium).

The CNCs were prepared at a pilot scale, the MCC was hydrolyzed with 60 wt% H_2_SO_4_ at 40 °C for 3 h, and the suspension was neutralized by the addition of K_3_PO_4_ and NaOH, followed by desalting with a ceramic ultrafiltration membrane (diameter 3 × 25 × length 1178 mm) (TAMI Industries, Nyon, France). Larger MCC particles were removed via a centrifugation step using a separation centrifuge from GEA Westfalia S1 1-01 (Oelde, Germany). The produced CNCs were concentrated at 55 °C, using a 0.5 bar steam pressure in a custom-made wiped film evaporator, a filling volume of 69 L, a maximum vacuum pressure of 1 bar, and a maximum temperature of 200 °C (GEA Canzler GmbH & Co. KG, Düren, Germany), and then lyophilized using an SP VirTis Genesis pilot freeze dryer 35 L (Barcelona, Spain) to obtain 1.4 kg of dry CNCs. The drying was performed with thermal treatment, primary drying, and secondary drying of −40 °C at 500 µbar, −40 °C to 25 °C for 44 h at 500 µbar, and 25 °C for 25 h at 80 µbar (condenser vacuum and temperature constant at 600 µbar and −45 °C), respectively. The freeze-dried CNCs are shown in Figure S1 in the Supplementary Materials.

The ChNCs were produced via sulfuric acid hydrolysis on a pilot scale by following the process reported in our earlier study [10]. Briefly, the shrimp chitin was hydrolyzed in a glass-lined reactor custom-made by Thaletec GmbH (Thale Germany) with 35 wt% H_2_SO_4_ at 60 °C for 2 h followed by centrifugation, dialysis, and homogenization to produce ChNCs. Isolated ChNCs had diameters and lengths ranging from 5 to 15 nm and 200 to 480 nm, respectively [10]. The supplied ChNCs (4 wt%) were then concentrated using a vacuum rotary evaporator to achieve the 18 wt% concentration.

### 2.2. Methods

#### 2.2.1. Blend Processing

The compositions of the prepared formulations are shown in Table 1. A masterbatch of PLA:PHB blend was prepared by ensuring the homogeneous mixing of biopolymers in a co-rotating twin-screw extruder (Leistritz 27E, Nuremberg, Germany) with a screw diameter (D) of 27 mm and a length/diameter ratio of 40. The screw speed was kept constant at 300 rpm, and the temperature profile was set in the range of 165–175 °C. The composition of the blend was preliminarily defined, and PLA and PHB were mixed in the specific ratio of 75:25 (wt%), respectively.

#### 2.2.2. Nanocomposite Processing

The PLA:PHB nanocomposite with OLA and CNCs was produced using conventional melt extrusion, in which the requisite amount of freeze-dried CNCs along with OLA was added to PLA-PHB blend using a twin-screw extruder (Leistritz 27E), at 300 rpm and the temperature profile in the range of 185–195 °C.

The PLA-PHB, OLA, and ChNCs were prepared via a liquid-assisted extrusion process using a co-rotating twin-screw extruder (Coperion W&P ZSK-18 MEGALab, Stuttgart, Germany). The PLA-PHB blend was fed using K-Tron gravimetric feeder (Niederlenz, Switzerland) attached to the extrusion. An aqueous suspension of ChNCs and OLA in the requisite amounts was added to produce nanocomposites. The suspension used for the liquid-assisted process was prepared by pre-dispersing a concentrated ChNC gel (18 wt%) in ethanol at a ratio of 1:5 (water:ethanol) using magnetic stirring for 2 h, followed by the addition of OLA. The suspension was ultrasonicated prior to extrusion and preliminarily heated at 50 °C with a heating plate before dosing.

#### 2.2.3. Film Blowing

**Figure 1:**
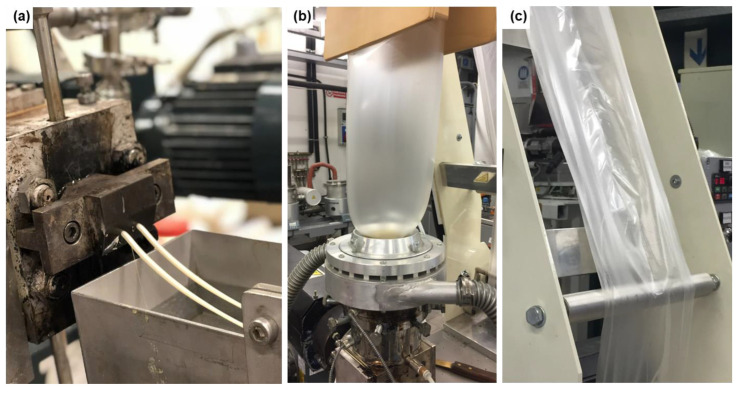
Material preparation process: (**a**) melt extrusion for blending PLA-PHB, OLA, and CNCs or ChNCs; (**b**) film blowing process; (**c**) the final nanocomposite bag.

The pellets from each formulation were dried at 80 °C for at least 4 h prior to extrusion. A MiniBlown 25 single-screw monolayer extrusion line (EUR.EX.MA Tradate, Italy) with a standard screw design (length 80 cm, diam 25 mm) attached with a circular opening die and chill rolls 380 for blow extrusion was used. For all the formulations, the velocity was set to 35 rpm and the throughput was 3.5 kg/h. The temperature profile was set at 170 °C in the barrel and 175 °C at the die. More than 2 kg of pellets was used for each formulation to ensure a steady film output. The facilities for the extrusion and film-blowing processes are shown in Figure 1.

The melt flow index (MFI) of the prepared pellets was measured as an indication of the blowability of the polymers using a modular melt flow device (Instron Ceast Model MF30, Pianezza, TO, Italy). The test was performed in accordance with the ASTM D1238 standard and EN ISO 1133 methods, at 190 °C with a 2.16 kg load. The procedure was repeated at least 3 times for each sample, and the average values are reported in grams per 10 min.

The thermal stability of the films was studied using the thermogravimetric analysis (TGA) Q500 model from TA Instruments (New Castle, DE, USA). The tests were performed in a temperature range from 50 to 800 °C in N_2_ atmosphere, with a heating rate of 10 °C/min (gas flow of 60 mL/min).

The thermal properties, glass transition temperature (T_g_), cold crystallization temperature (T_cc_), melting temperature (T_m_), and degree of crystallinity (Xc) were studied using the differential scanning calorimetry (DSC) Q800 model from TA Instruments (New Castle, DE, USA). A single heating scan was set from −50 to 210 °C, with a heating rate of 10 °C/min, to analyze Xc, which was calculated using the following Equation (1) [39]:

Xc = (ΔH_m_ − ΔH_cc_)/ΔH°_m_) × (100/w) (1)

where ΔH_m_ is the melting enthalpy, ΔH_cc_ is the enthalpy of cold crystallization, ΔH°m = 93 J/g for PLA, and w is the weight fraction of the measured film.

The morphology of the film fractured surface was studied by scanning electron microscopy (SEM) using a JEOL JSM-6460LV (JEOL, Tokyo, Japan) at an acceleration voltage of 5 kV. The samples were cryo-fractured in liquid nitrogen and the surface was sputter coated with platinum using an EM ACE200 Leica vacuum coater (Wetzlar, Germany) to avoid charging.

A Shimadzu AG conventional tensile tester (Kyoto, Japan) was used to test the mechanical properties of the materials. The samples were cut with a rectangular press mold with dimensions of 50 mm in length, 5.5 mm in width, and 44–56 µm in thickness. Prior to the testing, the samples were conditioned for 24 h at 25 °C in 50% RH. The test was carried out in the blowing direction (MD) using the following test parameters: a gauge length of 20 mm, strain rate of 2 mm/min, and 1 kN load cell. The tensile strength and elongation at break were directly available from the software, while the toughness and tensile modulus were calculated from the stress–strain data. The procedure was repeated for at least five samples and the average value is presented.

Oxygen permeability (OP), water vapor permeability (WVP), and biodegradability were carried out on the best material formulation based on the preliminary characterizations to determine the potential of the film used for food packaging.

OP and WVP tests were performed on the films using Multiperm 037 equipment (ExtraSolution, Fosciana, LU, Italy), according to the ASTM F2622-08 and ASTM F1249-20, respectively. The surface area of the square-formed films was 2 cm^2^. The films were previously conditioned for 6 h under a continuous flux of electronically controlled anhydrous nitrogen. This preliminary step is necessary to stabilize the specimens and to remove the oxygen already present inside the sample before the beginning of the test. The duration of this phase is strongly related both to the barrier properties and to the thickness of the material under testing. The greater the thickness of the specimen, the longer the conditioning phase will be. The test was performed at 23 °C and 50% RH relative humidity. The flux of the oxygen on the surface of the film was maintained at 13.5 mL/min on average. Two specimens were tested for each formulation.

The biodegradation test under composting conditions of the selected films was performed according to the modified standard ISO 20200. It corresponds to the physical fragmentation of the organic tested material into very small parts, due to the action of temperature, humidity, and the presence of micro-organisms. A specific composting environment was established in a perforated vessel reactor (30 × 20 × 10 cm^3^) by following the precise recipe specified in the standard, which includes 40 wt% sawdust, 30 wt% rabbit feed, 10 wt% ripe compost, 10 wt% corn starch, 5 wt% saccharose, 4 wt% corn seed oil, and 1 wt% urea (CAS N. 57-13-6, Merck Life Science, Rahway, NJ, USA). Around 5–6 g of each test specimen was (cut in 25 mm × 25 mm) buried at 4–6 cm depth in the vessel. The samples were incubated in a ventilated oven at 58 °C, and periodic and standard-determined monitoring of the weight of the samples was performed manually. Further, control was performed at 7, 14, 21, 28, and 45 days to control the level of disintegration. During the first week, a daily control was conducted by weighing the reactor vessel and, if necessary, adding water to restore the initial mass. After 30 days, the restoration of water was performed twice a week. After each extraction day, photographs of the films were taken for a qualitative evaluation of the physical degradation in compost over time.

## 3. Results

### 3.1. Material Processability

The MFI for PLA is 6.0 g/10 min, while the MFI of PLA-PHB blend and PLA-PHB-OLA masterbatch was 6.9 and 10.3 g/10 min, respectively. It appears that the addition of OLA affected the fluidity of the PLA-PHB blend. The higher MFI value in PLA-PHB-OLA can be attributed to the high polymer chain mobility, which decreases the melt viscosity and allows the polymer to flow easier [34]. On the other hand, the addition of CNCs and ChNCs resulted in a significant decrease in MFI to 6.4 g/10 min and 5.7 g/10 min in PLA-PHB-OLA-CNC and PLA-PHB-OLA-ChNC, respectively. This behavior is explained by the nanocrystals restricting the polymer chain mobility, resulting in an increase in polymer viscosity and melt strength [15,34].

The film blowing of the prepared material formulations was performed under stable processing conditions. The blowing process for the neat PLA-PHB blend was quite unstable due to bubble breaks and strain hardening caused by the poor melt strength and stiffness of the blend, resulting in wrinkles and creases with high thickness variation (Table 2) in the resultant film. The addition of OLA in PLA-PHB resulted in the formation of a smooth and stable process without any melt fracture during the blowing process of the PLA-PHB-OLA film because of the improved flowability. The addition of dry CNCs also caused difficulties in the blowing process and exhibited film with non-homogeneous thickness with some creases. Meanwhile, the film blowing from the nanocomposite pellet PLA-PHB-OLA-ChNC exhibited stable processing conditions resulting in a smooth and uniform film.

**Figure 2:**
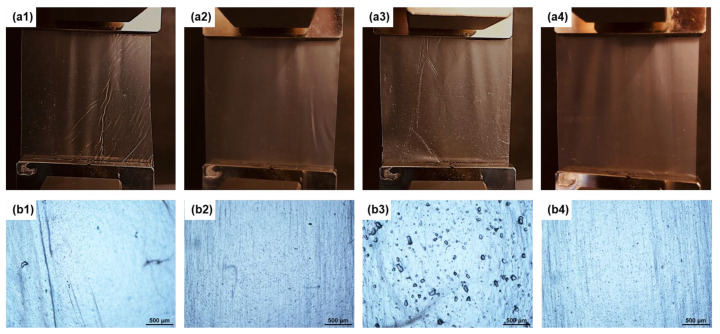
The visual appearance of PLA-PHB, PLA-PHB-OLA, PLA-PHB-OLA-CNC, and PLA-PHB-OLA-ChNC is shown in (**a1**–**a4**), respectively, and optical micrographs of PLA-PHB, PLA-PHB-OLA, PLA-PHB-OLA-CNC, and PLA-PHB-OLA-ChNC are shown in (**b1**–**b4**), respectively (scale bar 500 µm).

Figure 2 shows the visual appearance and microstructure of the prepared films. It was observed that most of the PLA-PHB blend film surface has wrinkle marks (strain hardening) in the blowing/machine directions as shown in Figure 2a1. The strain hardening occurs because of the stiffness of the polymer that is stretched in the machine direction during the process, causing fluctuation in internal bubble pressure and width of the bubble. However, this issue was resolved by the addition of OLA, resulting in an even, homogeneous, and smooth surface. On the other hand, the addition of CNCs to PLA-PHB-OLA resulted in a rough film with many white dots on the surface (Figure 2a3), which resembled large flakes when observed through a microscope (Figure 2b3). These particles are most likely CNC agglomerates that were not dispersed during nanocomposite preparation. The formation of these CNC agglomerates can be attributed to the freeze-drying step before the melt extrusion process, which resulted in an irreversible agglomeration that cannot be redispersed in the polymer matrix during the compounding process. Zaccone et al. [40] reported a similar phenomenon when dry ChNCs were fed into the PHB matrix, resulting in large ChNC agglomerates on the cast film surface. In contrast, liquid feeding of ChNCs in PLA-PHB-OLA-ChNC resulted in uniform, even, and smooth film with no evidence of nanomaterial agglomerates (Figure 2a4), indicating the positive effect of the liquid-assisted extrusion process, which preserves ChNCs in a dispersed state in the polymer matrix.

**Figure 3:**
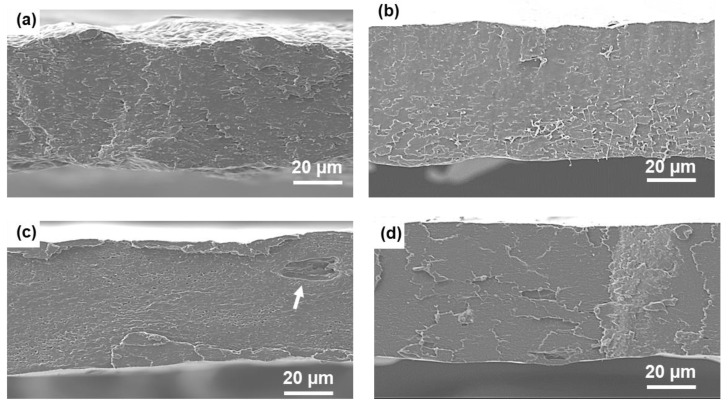
SEM images of the cryo-fractured surfaces of (**a**) PLA-PHB, (**b**) PLA-PHB-OLA, (**c**) PLA-PHB-OLA-CNC, and (**d**) PLA-PHB-OLA-ChNC.

The morphology of the blown film cryo-fractured surface and the dispersion of CNCs and ChNCs in PLA-PHB-OLA-CNC and PLA-PHB-OLA-ChNC, respectively, were evaluated using SEM, with the results shown in Figure 3. It can be seen that the PLA-PHB (Figure 3a) exhibited a brittle fractured surface, while PLA-PHB-OLA (Figure 3b) showed a smoother and more homogeneous surface due to the plasticizing effect of OLA. A fractured surface of the PLA-PHB-OLA-CNC blown film exhibited a void (white arrow in Figure 3c) which was most likely caused by CNC agglomerates present during sample preparation. However, PLA-PHB-OLA-ChNC (Figure 3d) exhibited a smooth fractured surface with no evidence of ChNC agglomerates, indicating that the liquid-assisted extrusion technique combined with the OLA resulted in good ChNC dispersion.

### 3.2. Material Properties

The thermal properties of PLA-PHB, PLA-PHB-OLA, PLA-PHB-OLA-CNC, and PLA-PHB-OLA-ChNC films were investigated using TGA and DSC. The influence of the addition of CNC and ChNC reinforcements on the thermal properties of the PLA-PHB-OLA matrix was investigated.

TGA results of the blown film materials are summarized in Table 3, and the thermograms are shown in Figure S2 in the Supplementary Materials. The thermal degradation of the materials exhibited similar behaviors, and the decomposition process took place in a stepwise manner; the first decomposition temperature represents the PHB degradation, while the second temperature is related to PLA degradation. T_onset_, the temperature corresponding to the initial 5 wt% weight loss, was above 200 °C for all the formulations, confirming the stability of the materials under the chosen process temperature without the risk of thermal degradation. The addition of OLA to the reference compound (PLA-PHB) did not change its thermal degradation behavior, as evidenced by the small differences in T_onset_ and T_max_ between PLA-PHB and PLA-PHB-OLA. The presence of the CNCs and ChNCs had a noticeable impact on both T_onset_ and T_max_, which delayed the beginning and maximum thermal decomposition process in both PLA-PHB-OLA-CNC and PLA-PHB-OLA-ChNC, resulting in a slight shift toward higher temperatures compared to reference material. These phenomena were also observed in our previous studies, in which ChNCs delayed the first degradation step of TEC and GTA plasticizer in PLA-TEC-ChNC [34] and PLA-PHB-GTA-ChNC [10], respectively. The inorganic residues of all the materials, obtained at the end of the test, were somewhat similar.

The thermal properties from the first heating DSC scan of each film are summarized in Table 4, while the thermograms are shown in Figure S3 in the Supplementary Materials. It is observed that all formulations followed a nearly identical pattern. Furthermore, all the films exhibited a single peak for glass transition temperature (T_g_) and melting temperature (T_m_), which corresponds to PLA. The single melting peak, according to Zhang and Thomas [8], suggests a higher degree of miscibility between PLA and PHB. In addition, it is evident that the addition of OLA had no significant effect on T_g_ and T_m_ but shifted the T_cc_ value toward lower temperatures (from 116 to 104 °C). This decrease in T_cc_ was primarily attributable to an increase in chain mobility because of OLA, which allows the material to recrystallize during the heating process, as evidenced by the increase in the Δ_cc_ value of PLA-PHB-OLA. However, the presence of CNC and ChNC restricted the chain mobility and consequently increased the T_g_ and T_cc_ values. The addition of OLA decreased the degree of crystallinity (Xc) in PLA-PHB-OLA, while the nanocomposite films were slightly more crystalline than the control (PLA-PHB), indicating an increase in the nucleation of spherulites due to the CNCs and ChNCs, which is consistent with our previous findings. The combination of ChNC and OLA produced a higher crystallinity degree, confirming its ability to act as a nucleating agent. The higher crystallinity results in higher melt enthalpy (ΔH_m_) of the nanocomposite.

In summary, the presence of CNCs and ChNCs in the formulations results in greater thermal stability, indicating that the presence of CNCs or ChNCs along with OLA has a reinforcing effect on the PLA-PHB-OLA-ChNC film.

The influence of processing and the effect of CNC and ChNC reinforcements on mechanical properties were investigated using a tensile test. The blown films were tested in the blowing direction (MD), and the mechanical properties, including Young’s modulus, tensile strength, elongation at break, and work of the fracture, are summarized in Table 5, while the representative stress–strain curves for all the films are shown in Figure S4 in the Supplementary Materials.

The results indicate that blown film from the PLA-PHB blend showed reduced mechanical properties compared to the neat PLA properties reported by the manufacturer. It is possible that the wrinkled and uneven surface of the PLA-PHB films, caused by an unstable film-blowing process, hindered good stress transfer during the tensile test. This may explain the relatively lower mechanical properties. The addition of OLA to the PLA-PHB blend had minimal impact on the tensile strength (σ_max_) and Young’s modulus, whereas the flexibility of the material significantly increased to 58%, which is 29 times higher than that of the PLA-PHB blend, due to the plasticizing effect [10,34,42], resulting in a tougher material.

The addition of 1 wt% CNCs had a negative influence on the mechanical properties and resulted in a significant decrease in elongation at break (from 57.6% to 11.2%) in the PLA-PHB-OLA-CNC nanocomposite film. The decrease in flexibility of nanocomposites has been attributed to the presence of a large number of CNC agglomerates that act as stress concentrators and promote the propagation of interface-generated defects, resulting in film failure [43], which is in good agreement with optical microscopy.

However, the addition of ChNCs resulted in a substantial improvement in the mechanical performance of the PLA-PHB blend. The tensile strength increased from 24 MPa (PLA-PHB-OLA) to 37 MPa (54%), the modulus increased from 2.7 to 2.9 GPa, and the elongation at break increased from 58% to 71%, showing the best properties among all blown films. In addition, when compared to the PLA-PHB blend, the nanocomposite films were found to be more stretchable (35 times), as well as tougher (10 times); these are important properties, especially for film blowing. The reason that the addition of the ChNCs can improve the PLA-PHB blend behavior is expected to be related to the homogeneously dispersed ChNCs in PLA-PHB matrix, as well as strong interfacial adhesion and formation of a rigid ChNC percolating network within the polymer matrix which would facilitate an effective stress transfer from the polymer matrix to rigid ChNCs network, resulting in an improvement in the strength and toughness of polymer nanocomposites [24,44].

Films for food packaging are required to maintain their integrity in order to withstand the stress that occurs during shipping, handling, and storage [45]. In comparison to petroleum-based polymers such as polypropylene (PP) and low-density polyethylene (LDPE), which are frequently used in packaging applications, PLA-PHB-ChNCs nanocomposite films were shown to be stiffer, while maintaining comparable tensile strength and stretchability. Therefore, PLA-PHB-OLA-ChNC nanocomposite film, produced with liquid-assisted extrusion and the film-blowing process, could be considered the best formulation and process for food packaging applications.

The oxygen and water vapor barrier properties of PLA-PHB-OLA and PLA-PHB-OLA-ChNC films were investigated to determine the impact of the ChNCs, and the results are summarized in Table 6. Because the tests were conducted on blown films, the permeation parameter is critical and needs to be considered for both the OTR and WVTR to obtain values that are comparable between the two materials. The OTR value for PLA-PHB-OLA film was 1226 cc/m^2^ 24 h, which is approximately 34% lower than the OTR for pure PLA. The decrease in OTR value can be attributed to the increase in crystallinity caused by blending with a high-crystallinity polymer (PHB). The addition of ChNCs has a further two-fold effect on the OTR and reduces it from 1226 to 630 cc/m^2^ 24 h, which could be explained by a higher degree of crystallinity in the nanocomposites as well as inherent barrier properties of the ChNCs, leading to an increased tortuous path and consequently slowing down the permeation rate. It is worth noting that the OTR value of the final nanocomposite film is noticeably lower than that of commercial polymers that are commonly used for food packaging applications, such as low-density polyethylene and polypropylene. While the addition of ChNCs decreased the gas permeability, an opposite behavior is seen in water vapor permeability. Indeed, this value for PLA-PHB-OLA-ChNC is higher than that for the corresponding PLA-PHB-OLA film. The hydrophilic nature of ChNCs, which attracts more water molecules and leads to an increase in WVTR value, is one of the possible explanations for this phenomenon.

**Figure 4:**
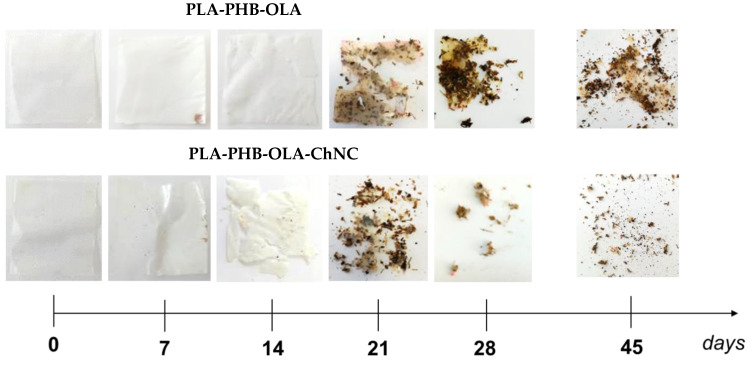
Disintegration of produced nanocomposites under composting conditions: pictures of specimens at different test days.

Figure 4 displays the visual appearance of the PLA-PHB-OLA and PLA-PHB-OLA-ChNC films after different time periods of disintegration in composting conditions. It is possible to observe the beginning of the disintegration of both films after 14 days as an initial cracking of the tested samples is visible. After 21 days, however, both materials exhibited strong fragmentation and a significant change in color, transitioning from a transparent appearance to yellowish color of the disintegrated particles. Nevertheless, the presence of ChNCs in PLA-PHB-OLA formulation seems to accelerate the disintegration process. These findings are in good agreement with the study reported by Arrieta et al. [47], where chitosan was found to accelerate the disintegration process of the PLA-PHB blend. Both materials are completely disintegrated after 45 days, which corresponds to the minimum amount for compliance with the adopted standard. In addition, the fragments of PLA-PHB-OLA-ChNC visible to the naked eye are smaller than the PLA-PHB-OLA ones.

The accelerating behavior of ChNCs in disintegration may be attributed to the sensitivity of this nanofiller to humidity and water vapor. These results can be linked to the poor water vapor barrier properties of the nanocomposite film. In fact, Asri et al. [48] demonstrated this kind of correlation, reporting that nanocomposites with higher water vapor permeability also exhibit better disintegration behavior. This phenomenon can be mainly explained because hydrophilic nanocomposites easily allow the microorganism to penetrate the material, accelerating the breakdown of polymeric chains and increasing the degradation rate.

## 4. Conclusions

A pilot-scale approach to produce PLA-PHB-based nanocomposite films reinforced with CNCs and ChNCs intended for food packaging was studied. A masterbatch of PLA-PHB blend with a 75:25 ratio was prepared using twin-screw extrusion followed by the compounding of the nanocomposites and film-blowing process. A nanocomposite with CNCs was made by feeding dry CNCs in the compounding process, while a nanocomposite with ChNCs was produced via the liquid-assisted extrusion method.

The nanocomposite produced via liquid-assisted extrusion exhibited a stable blowing process with smooth and homogeneous film, whereas the addition of dry CNCs resulted in agglomerates and non-homogeneous and rough surfaces in the produced blown film. The addition of both CNCs and ChNCs improved the thermal stability of nanocomposite films. The combination of OLA and well-dispersed ChNCs showed a reinforcing effect and simultaneously increased the tensile strength and stiffness compared to the biopolymer films and nanocomposite with CNCs.

The ChNC nanocomposite films were found to have better strength (37%), flexibility (35-fold), and toughness (10-fold) and comparable modulus when compared with the PLA-PHB blend.

The synergic effect of better-dispersed ChNCs with the assistance of OLA resulted in increased crystallinity and, thereby, an improvement in the oxygen barrier performance when compared to neat PLA.

In addition, the disintegration behavior under composting conditions of the best films was studied, and the nanocomposite films with ChNCs showed accelerated degradation.

The findings of this research indicate that the multifunctional PLA-PHB-OLA-ChNC film prepared via liquid-assisted extrusion and film blowing has great potential as a flexible film and opens a new perspective for its industrial application as short-term food packaging.

## Figures and Tables

**Table 1:** Compositions of the biopolymer blends and nanocomposites are given in weight %.

Materials	PLA-PHB (75-25)	OLA	CNC	ChNC
PLA-PHB	100	-	-	-
PLA-PHB-OLA	96	4	-	-
PLA-PHB-OLA-CNC	95	4	1	-
PLA-PHB-OLA-ChNC	95	4	-	1

**Table 2:** The thickness of blown films produced from the compounded materials.

Materials	Film Thickness (µm)
PLA-PHB	52 ± 12
PLA-PHB-OLA	48 ± 5
PLA-PHB-OLA-CNC	48 ± 8
PLA-PHB-OLA-ChNC	47 ± 3

**Table 3:** TGA of the blown films (* T_onset_ is initial 5% weight loss).

Materials	T_onset_ * (°C)	T_max_ (°C)		Residue (%)
		PHB	PLA	
PLA-PHB	282	293	364	0.43
PLA-PHB-OLA	282	295	362	0.47
PLA-PHB-OLA-CNC	288	299	366	0.53
PLA-PHB-OLA-ChNC	289	302	369	0.50

**Table 4:** Thermal properties of blown film materials obtained from DSC first heating scan.

Materials	T_g_ (°C)	T_cc_ (°C)	ΔH_cc_ (J/g)	T_m_ (°C)	ΔH_m_ (J/g)	Crystallinity (X_C_%)
PLA-PHB	52	116	6.6	150	16.0	10.2
PLA-PHB-OLA	51	104	12.6	151	17.2	5.2
PLA-PHB-OLA-CNC	57	117	7.0	151	17.6	12.1
PLA-PHB-OLA-ChNC	56	110	9.0	146	21.2	13.8

**Table 5:** Mechanical properties of the blown films tested in the blowing direction compared with PLA, LDPE, and PP.

Materials	Strength (MPa)	Modulus (GPa)	Elongation at Break (%)	Toughness (MJ/m^3^)
PLA-PHB	26.8 ± 3.4	2.9 ± 0.1	2 ± 1	1.8 ± 0.8
PLA-PHB-OLA	24.3 ± 2.1	2.7 ± 0.3	58 ± 6	13.6 ± 0.8
PLA-PHB-OLA-CNC	23.4 ± 0.9	2.8 ± 0.3	11 ± 0	4.9 ± 0.7
PLA-PHB-OLA-ChNC	36.8 ± 2.8	2.9 ± 0.1	71 ± 9	19.8 ± 1.3
PLA (data sheet)	53	3.5	6	-
LDPE [41]	8.3–31.4	0.17–0.28	100–650	-
PP [41]	31–41.4	1.14–1.55	100–600	-

**Table 6:** Oxygen and water vapor barrier properties of produced materials.

Materials	Oxygen Permeability		
	OTR (cc/m^2^ 24 h)		Permeation (cc µm /m^2^ 24 h)
PLA-PHB-OLA	1226		67,244
PLA-PHB-OLA-ChNC	630		25,178
PLA [10]	1853		-
PE [46]	3374		-
PP [46]	1589		-
	Water vapor permeability		
Materials	WVTR (cc/m^2^ 24 h)	Permeance (g/m^2^ 24 h mmHg)	Permeability (g mm/m^2^ 24 h mmHg)
PLA-PHB-OLA	33.8	2.4	0.12
PLA-PHB-OLA-ChNC	93.5	6.7	0.18

## Data Availability

Data are available from the corresponding author upon reasonable request.

## Supplementary Materials

The following supporting information can be downloaded at: https://www.mdpi.com/article/10.3390/polym14235104/s1, Figure S1: (a) SEM image of cellulose nanocrystals before freeze drying; (b) freeze-dried cellulose nanocrystals; (c) optical microscopy of dried nanocrystals showing agglomeration. Figure S2: TGA thermogram of blown films. Figure S3: DSC thermogram of the blown films. Figure S4. Tensile stress–strain graph of blown films in blowing direction.
