# Supplementary material for: Improvement of Poly(lactic acid)-Poly(hydroxy butyrate) Blend Properties for Use in Food Packaging: Processing, Structure Relationships
Source: Polymers (Basel). 2022 Nov 24;14(23):5104. doi: 10.3390/polym14235104 (PMC9736990; doi:10.3390/polym14235104)
Supplement: Supplementary file 1 [file polymers-14-05104-s001.zip › polymers-2016273-supplementary.pdf]

Supplementary Materials

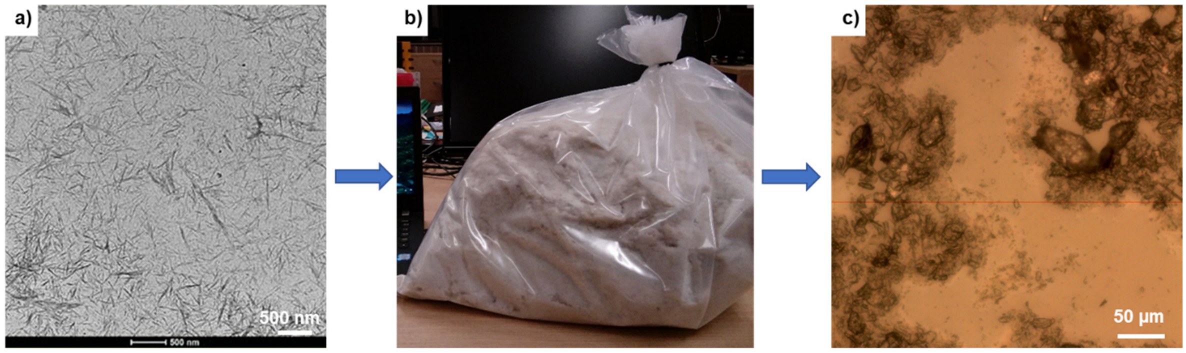

Figure S1. a) SEM image of cellulose nanocrystals before freeze-dried b) Freeze dried cellulose nanocrystals c) optical microscopy of dried nanocrystals showing agglomeration.

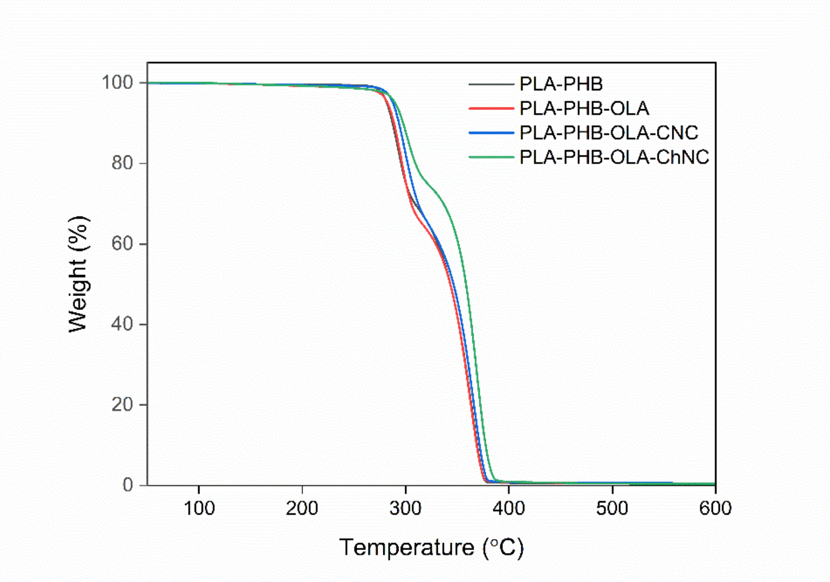

Figure S2. TGA thermogram of the blown films.

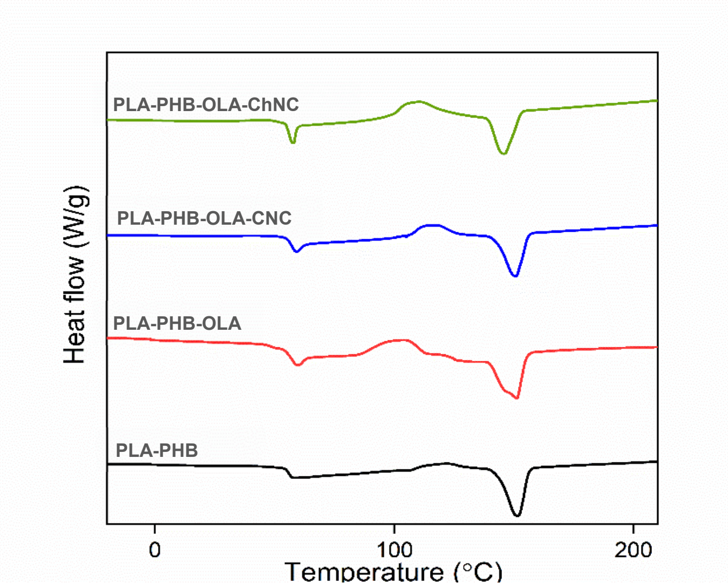

Figure S3. DSC thermogram of the blown films.

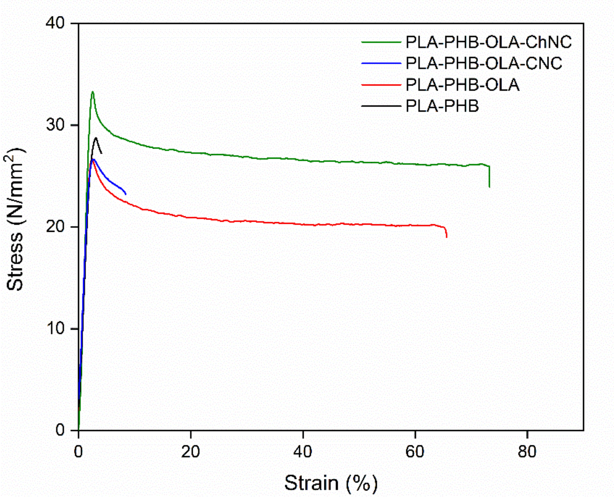

Figure S4. Tensile stress-strain graphs of blown films in blowing direction.
